# Plasmalogens in bacteria, sixty years on

**DOI:** 10.3389/fmolb.2022.962757

**Published:** 2022-11-14

**Authors:** Howard Goldfine

**Affiliations:** Department of Microbiology, Perelman School of Medicine, University of Pennsylvania, Philadelphia, PA, United States

**Keywords:** bacteria, evolution, genetics, plasmalogen, biosynthesis

## Abstract

The presence of plasmalogens in bacteria has been known for 60 years. The recent discovery of two genes encoding reductases that convert diacyl lipids to 1-alk-1′-enyl 2-acyl lipids has confirmed the derivation of plasmalogens from the corresponding diacyl lipids in bacteria. These genes are widely distributed in anaerobic and in some facultatively anaerobic bacteria. Plasmalogens evolved very early in the history of life on earth. Their persistence during eons of evolution suggests that they play a fundamental role in living organism. The phase behavior of plasmalogens and their conformation in membranes is discussed.

## Introduction

An understanding of specific roles played by individual lipid types has expanded greatly in the past several decades. Specifically, the importance of a balance between lipids that assemble into bilayers readily, as opposed to those that easily transition to non-bilayer assemblages has become widely appreciated. In addition, specific roles played by unusual lipid species have been uncovered. Plasmalogens, with their alk-1′-enyl ether chain are different from the more common all acyl lipids chemically, in their phase behavior and in their three-dimensional structures in membranes. The recent discovery of the genes for plasmalogen synthesis in bacteria has confirmed their direct formation from the corresponding diacyl lipids. In this review, the presence of plasmalogens in many anaerobic and some facultatively anaerobic bacteria will be discussed. These lipids evolved very early in the history of life on earth. Their persistence suggests that they play a fundamental role in these living cells.

## Distribution of plasmalogens in bacteria

**FIGURE 1:**
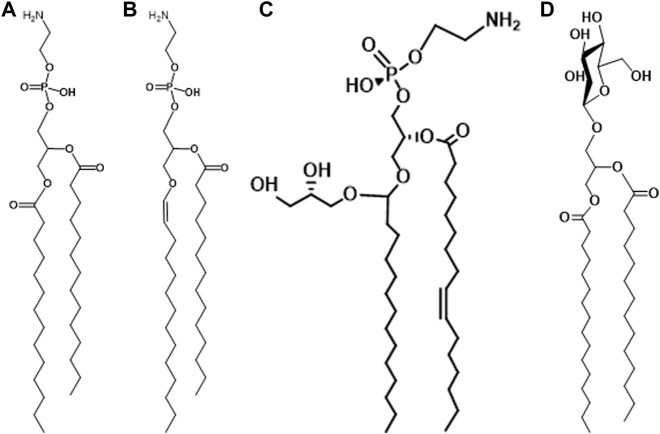
**(A)** phosphatidylethanolamine; **(B)** plasmenylethanolamine; **(C)** glycerolacetal of plasmenylethanolamine; **(D)** glycosyldiacylglycerol.

The presence of plasmalogens in anaerobic bacteria was discovered in the early 1960s. The first report, by Allison *et al.* concerned the polar lipids of *Ruminococcus flavefaciens* (Allison et al., 1962). This anaerobic, Gram-positive species is related to *Clostridium*. The ratio of aldehydes released from plasmalogens to phosphorus was reported to be 0.56–0.80 suggesting that most of the polar lipid was plasmalogen. At the same time, Wegner and Foster (Wegner and Foster, 1963) were exploring the lipids of *Bacteroides succinogenes* and reported a similar high ratio of aldehyde to phosphorus. The major polar lipids are phosphatidylethanolamine and plasmenylethanolamine in this organism (Figures 1A,B).

The first reports on the presence of plasmalogens in *Clostridium* described the polar lipids of *Clostridium butyricum* ATCC 6015 (Goldfine, 1964; Baumann et al., 1965). The strain was subsequently reclassified as *Clostridium beijerinckii* because one of its major phospholipids, phosphatidyl-N-methylethanolamine (PME), is not present in *C. butyricum* (Goldfine, 1962; Johnston and Goldfine, 1983). The major lipids: phosphatidylethanolamine (PE), phosphatidyl-N-methylethanolamine (PME), phosphatidylglycerol (PG) and cardiolipin (CL), are all present as all acyl and 1-alk-1′-enyl, 2-acyl species. An additional ether lipid is present among the phospholipids and was later identified as a glycerol acetal of plasmenylethanolamine (Figure 1C) (Matsumoto et al., 1971). In *C. butyricum* all of the above lipid species except for PME are present (Johnston and Goldfine, 1983). As in *C. beijerinckii*, all the major lipids are present as all acyl and alk-1′-enyl, acyl species. Many Gram-positive bacteria have glycosyldiradylglycerols (Figure 1D) and a substantial portion of these is often 1-alk-1′-enyl 2-acyl lipids. There can be as many as four sugars, but in most cases, they have not been structurally identified (Guan and Goldfine, 2021).

In 1969 Kamio *et al.* published a survey of plasmalogens and saturated ether lipids in bacteria. The aldehyde/P ratio ranged from a low of 0.04 in *Clostridium perfringens* to a high of 1.04 in *Peptostreptococcus elsdenii,* now *Megasphaera elsdenii* (Kamio et al., 1969; Rogosa, 1984). Most species had very low levels of saturated ethers, which are characteristic of Archaea (Koga and Morii, 2005). Since then, there have been several reviews on plasmalogen distribution in bacteria (Goldfine and Johnston, 2005; Rezanka et al., 2012; Guan and Goldfine, 2021; Vitova et al., 2021).

## Plasmalogen biosynthesis--the anaerobic pathway

The elucidation of the eukaryotic biosynthetic pathway for plasmalogen biosynthesis in the early 1970s revealed a requirement for molecular oxygen to affect the desaturation of a saturated ether precursor (Snyder, 1972; Snyder, 1999). It became apparent that nature has evolved two mechanisms for formation of lipids containing an alk-1′-enyl ether bond (Goldfine, 2010). Since early life evolved in an anaerobic environment, the anaerobic pathway is presumably the more ancient. At that time in Earth’s history, respiration had not yet evolved. When respiration appeared as the concentration of O_2_ in the earth’s atmosphere increased, reactive oxygen species (ROS) were produced as a by-product of respiration. Plasmalogens are highly sensitive to ROS and thus became undesirable membrane constituents. Aerobic and facultative organisms were able to survive and reproduce with lipids containing only acyl ester lipids (Goldfine, 2010).

As described in other articles in this series, the eukaryotic pathway to plasmalogens begins with the synthesis of a saturated ether lipid formed first by the acylation of dihydroxyacetone phosphate (DHAP) catalyzed by glycerone phosphate acyl transferase (GNPAT). A long-chain alcohol then displaces the *sn*-1 acyl chain to form 1-*O*-alkyl-2-hydroxy-glycerol-3-P catalyzed by alkylglycerone phosphate synthase (AGPS) on the luminal side of the peroxisomal membrane (da Silva et al., 2012). After acylation of the sn-2 hydroxyl group to form 1-O-alkyl-2-acyl glycerol-2-P and the subsequent formation of 1-O-alkyl-2-acyl-glycerol-P-ethanolamine, there is an oxygen-dependent desaturation of the 1-O-alkyl chain to produce plasmenylethanolamine (PlsE) (Figure 1B) (Gallego-García et al., 2019; Werner et al., 2020).

**FIGURE 2:**
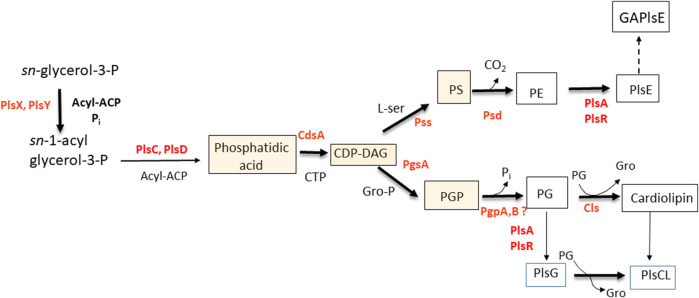
Pathway for the biosynthesis of plasmalogens in *Clostridium butyricum.* The shaded boxed intermediates have essentially no plasmalogen species in *C. novyi* and *C. tetani.* Abbreviations: ACP, acyl carrier protein; CL, cardiolipin; DAG, diacylglycerol; GAPlsE, glycerolacetal of plasmenylethanolamine; PE, phosphatidylethanolamine; PG, phosphatidylglycerol; PlsE, plasmenylethanolamine; PlsG, plasmenylglycerol, PS, phosphatidylserine.

It has been clear for several decades that the formation of plasmalogens in anaerobes succeeds the formation of the cognate diacylglycerol-phospholipids. The earliest studies indicated a precursor-product relationship between PE and PlsE (Baumann et al., 1965). DHAP was eliminated as a precursor of plasmalogens in *Clostridium beijerinckii* (Hill and Lands, 1970), *Veillonella parvulla*, and *Megasphaera elsdenii* (Prins and Van Golde, 1976). DHAP is also not a precursor to plasmalogens in two anaerobic protozoa, indicating that the dividing line is anaerobic vs. aerobic, rather than prokaryotic vs. eukaryotic (Prins and Van Golde, 1976). Long-chain alcohols were not utilized for plasmalogen biosynthesis in bacteria, but long-chain aldehydes were (Goldfine and Hagen, 1972). The formation of plasmalogens in bacteria from diacylphospholipids received further support in later experiments with whole cells in which the decarboxylation of phosphatidylserine (PS) was inhibited. The mostly diacyl PS that accumulated was rapidly converted to PE followed by PlsE when *the* block was removed (Koga and Goldfine, 1984). Mass spectrometric analysis showed that precursors of phospholipids in bacteria including: phosphatidic acid, CDP-diacylglycerol, and PS were essentially devoid of alk-1′-enyl ether species (Johnston et al., 2010; Guan et al., 2011). In more recent experiments, PS containing odd-chain fatty acids (C_17_), not present in *C. beijerinckii*, was taken up by growing cells, it was then decarboxylated to form PE, which was transformed to PlsE containing the identical odd-chains (Figure 2) (Goldfine, 2017).

The long-sought bacterial genes for the formation of plasmalogens by direct reduction of the *sn*-1 acyl chain were identified in *Clostridium perfringens* (Jackson et al., 2021). The genes *plsA* and *plsR* encode reductases that sequentially convert the acyl ester to an alk-1′-enyl ether. As expected, these genes are present in many Firmicutes, which includes Costridiales, Veillonellales, Tissierallales, Selenomonadeles, Erysipelotrichales and Acidaminococcales. Among Actinobacteria, these genes are also present in Propionibacteriales, Eggerthellales, Coriobacteriales, Bifidobacteriales and Actinomycetales. Some groups of Proteobacteria have orthologs of these genes (Jackson et al., 2021).

Unexpectedly, these studies have revealed the presence of *plsA* and *plsR* orthologs in facultatively anaerobic bacteria such as *Listeria*, which appear to be active when the cells are grown under anaerobic conditions, confirming earlier reports of the presence of plasmalogens in *L. monocytogenes* and *Enterococcus* faecalis (Farizano et al., 2019). A TetR/AcrR-like transcriptional regulator (EF1326) is present directly upstream of the *pls* gene in *E. faecalis* (EF1327) and other facultative anaerobes. This potentially O_2_-sensing transcriptional regulator is not present in strict anaerobes. These findings open the question of the need for plasmalogens under anaerobic, but not aerobic conditions, which is discussed below.

## The conformation of plasmalogens and their biophysical properties

With respect to gel to liquid phase transition temperatures, the presence of the *sn*-1-vinyl ether bond has a modest, but measurable effect, lowering the transition temperature 4–6°C (Goldfine et al., 1981; Lohner et al., 1984). A more dramatic effect is seen in the transition from a lamellar to non-lamellar phase. The lamellar to hexagonal II (L → H) for a semi-synthetic PlsE was 30°C compared to 68°C for the diacyl form (Lohner et al., 1984). In biological membranes rich in PlsE, the ability to prevent this transition, which is incompatible with normal membrane function, is necessary. This is usually accomplished by the presence of negatively charged lipids such as PG, CL or PS, and in the case of solvent-producing bacteria, by the presence of a glycerolacetal of PlsE (Figure 1C) (Johnston and Goldfine, 1985; Goldfine et al., 1987).

Diacyl PE molecules in model membranes display a bend at C-2 in the *sn*-2 acyl chain so that the first and second carbon atoms lie essentially parallel to the bilayer plane (Hitchcock et al., 1974). In contrast, this chain in plasmalogens appears to be perpendicula*r* to the bilayer surface (Malthaner et al., 1987a; Malthaner et al., 1987b; Han and Gross, 1990). A molecular dynamics simulation study supports the closer packing of the proximal regions, which results in thicker lipid bilayers. These simulation studies have shown that in plasmalogens the vinyl-ether linkage increases the ordering of the sn-1 chain and the sn-2 acyl chains in PlsCs and PlsEs (Rog and Koivuniemi, 2016; Koivuniemi, 2017). Taken together, these studies indicate tighter packing of plasmalogens in biomembranes, consistent with studies showing that artificial membranes with plasmalogens are less permeable to small molecules (Chen and Gross, 1994; Zeng et al., 1998).

## Functions of plasmalogens

Animals including humans require plasmalogens for normal development and function. As discussed in other articles in this series, humans lacking plasmalogens suffer from Rhizomelic chondrodysplasia punctata (RCDP), a condition that impairs normal development of the bones in the upper arms and thighs (rhizomelia). Other consequences of the absence of plasmalogens include intellectual disability, cataracts and heart defects (Braverman and Moser, 2012).

The recent discovery of an operon in bacteria that encodes two reductases needed for plasmalogen biosynthesis in bacteria has opened the field to discovery of the effects of plasmalogen deficiency in bacteria. As of now, there have been no reports on the effects of these mutations on bacterial structures and physiology. In the past, plasmalogen-deficient strains of *Megasphaera elsdenii* were isolated after serial subculture. Strains of this species were isolated in which the normal ratio of plasmalogen to lipid phosphorus was reduced from 0.8 to less than 0.05. Only small changes in morphology and end-products of fermentation were reported. A notable change was a large decrease in saturated fatty acids in the major lipids (Kaufman et al., 1988; Kaufman et al., 1990). Physical studies that compared the membranes and lipids of the wild type and the plasmalogen-deficient strain revealed somewhat lower ordering of the phospholipids compared to the wild type. Both ^31^P NMR and X-ray diffraction revealed that lipids from the wild-type strain underwent transition from the bilayer arrangement to a hexagonal phase, beginning at 30°C. Phospholipids from plasmalogen-deficient strains appeared to form a relatively stable lamellar phase. Thus, the presence of plasmalogens promoted the formation of non-lamellar phases as found in studies with model membranes (Lohner et al., 1984).

Examination of the polar lipids of *Clostridium tetani* ATCC 10779, the parent strain of strain E88, which was the first *C. tetani* strain to have its genome sequenced, revealed that it did not have plasmalogens. Analysis of several other *C. tetani* strains showed that they all had mixtures of all acyl and plasmalogen polar lipids including PE, PG, CL and N-acetylglucosaminyl diradylglycerol (Johnston et al., 2010). Strain ATCC 10779 was used for production of tetanus toxoid, and it is possible that the ability to form plasmalogens was lost during serial passage. No physical studies were done on the polar lipids of the wild-types and the plasmalogen-deficient strain ATCC 10779.

In general, the results with natural membrane lipids from *M. elsdenii*, support previous work with semisynthetic plasmalogens and those isolated from fatty acid auxotrophic bacteria. The presence of plasmalogens results in closer packing and destabilization of the lamellar organization. As Koivuniemi has pointed out, it seems probable that plasmalogens play an important role in exosome fission, but this is an unlikely general role in bacteria. More likely is the tighter packing they provide resulting from the absences of a bend at the C-2/C-3 carbons of the *sn*-2 acyl chains. The retention of the ability to synthesize plasmalogens in anaerobic and facultatively anaerobic species over eons of evolution speaks to a generalized function. One characteristic of fermentative organisms is the production of acids and expulsion of protons into the extracellular space. This is true for saccharolytic and proteolytic species of *Clostridium* (Holdeman et al., 1977). As the surrounding medium acidifies, the concentration of protons increases and it is imperative that protons do not return to the cytoplasm. Hence, membranes containing plasmalogens that are less permeable than those containing diacyl phospholipids alone would be favored. As the concentration of oxygen in the atmosphere increased, the development of respiration disfavored fermentation and the concomitant production of acids. As noted above, respiration produces reactive oxygen species, which are destructive of plasmalogens. As organisms that are more complex evolved, plasmalogens again appeared, but in animal cells, they were formed by an oxygen-dependent mechanism. It is instructive to note that in higher organisms, plasmalogens are concentrated in conductive tissues such as the heart and central nervous system, where their enhanced barrier function plays an important role.

## Abbreviations

CL, cardiolipin; DHAP, dihydroxyacetone phosphate; PE, phosphatidylethanolamine; PG, phosphatidylglycerol; PlsE, plasmenylethanolamine; PME, phosphatidyl-N-methylethanolamine; PlsG, plasmenylglycerol; PS, phosphatidylserine.
